# Establishment of an endoplasmic reticulum stress-related signature predicting outcomes of gastric adenocarcinoma patients

**DOI:** 10.3389/fgene.2022.944105

**Published:** 2022-09-06

**Authors:** Meiyuan Gong, Jingtao Wang, Wenfang Gao, Qian Liu, Jiaxing Chen, Guojun Wang, Qi Zhang

**Affiliations:** ^1^ State Key Laboratory of Esophageal Cancer Prevention and Treatment, School of Pharmaceutical Sciences, Zhengzhou University, Zhengzhou, China; ^2^ Department of Gastrointestinal Surgery, The First Affiliated Hospital of Zhengzhou University, Zhengzhou, China

**Keywords:** gastric adenocarcinoma, prognostic signature, endoplasmic reticulum stress, immune filtration, immune checkpoint inhibitors

## Abstract

**Background:** Gastric adenocarcinoma (GAC) is a common clinical malignancy with a poor prognosis. Endoplasmic reticulum (ER) stress plays important roles in the progression, immune filtration, and chemoresistance of cancers. However, whether ER stress-related gene signatures can predict the prognosis of GAC patients remains unknown.

**Methods:** GAC patient RNA-seq data downloaded from The Cancer Genome Atlas and gastric cancer patient microarray data from Gene Expression Omnibus datasets were analyzed using LASSO regression to construct an ER stress-related signature. Survival analysis, time-dependent receiver operating characteristic (ROC) curves, and Cox regression analysis were used to verify the efficacy of the signature. Immune infiltration, somatic mutation, immune checkpoint, and copy number variation analyses were utilized to explore the potential biological significance of the signature.

**Results:** In the present study, eight ER stress-related gene signatures were constructed. Survival analysis showed that patients in the high-risk group had a significantly worse prognosis. The area under the time-dependent ROC curves was 0.65, 0.70, and 0.63 at 1, 3, and 5 years, respectively, in the training cohort. Cox regression analysis showed that the signature is an independent prognostic factor. To predict GAC patients’ prognosis meeting individual needs, a nomogram was constructed with good accuracy. In addition, gene set enrichment and immune infiltration analyses showed that the ER stress-related signature is associated with cancer-related pathway activation and an immunosuppressive tumor microenvironment in GAC.

**Conclusion:** In the current study, we established an ER stress-related signature. This prognostic signature has good predictive power and could facilitate the development of novel strategies for the clinical treatment of GAC.

## Introduction

Gastric cancer is one of the most common malignancies worldwide, ranking fifth in the number of new cases of all cancer types in 2020 (Sung et al., 2021). The most common histological type of gastric cancer is gastric adenocarcinoma (GAC), and China accounts for 40% of new cases each year globally (Ajani et al., 2017). Despite great progress in the treatment of GAC in recent years, the 5-year overall survival (OS) remains low (Joshi and Badgwell, 2021; Zheng et al., 2021). Therefore, it is important to identify a novel prognostic signature for predicting the prognosis of GAC patients and instructing rational treatment.

The endoplasmic reticulum (ER) is the main site for protein synthesis and folding in eukaryotic cells and plays an important role in maintaining intracellular homeostasis (da Silva et al., 2020). However, external stimuli such as hypoxia, reactive oxygen species, and drug exposure can disrupt the homeostasis of the ER, triggering protein misfolding and accumulation of proteins, ultimately leading to ER stress (Chen and Cubillos-Ruiz, 2021). Interestingly, ER stress can trigger the unfolded protein response (UPR), a protective mechanism that induces a series of transcriptional and translational changes to promote cell adaptation (Roy and Kumar, 2019). If these corrective measures are inadequate to restore homeostasis, various ER sensors actively signal cell destruction (Oakes and Papa, 2015).

ER stress plays an important role in promoting tumor growth, the tumor immune microenvironment, and chemoresistance. The uncontrolled rapid growth of cancer cells and the hypoxic, nutrient-poor tumor environment result in a state of ER stress in tumor cells (King and Wilson, 2020). Induction of the UPR by ER stress enables cells to adapt to adverse environmental conditions and promotes tumor progression (Madden et al., 2019). In addition, the adverse effects of tumor cells on immune cells can disturb the ER homeostasis of immune cells, thus hindering effective antitumor immunity (Song and Cubillos-Ruiz, 2019). Various factors secreted by tumor cells can induce ER stress in macrophages, which is conducive to the survival of tumor cells (Di Conza et al., 2021). In addition, multiple ER stress-related genes are upregulated in tumor cells to promote chemoresistance, for example, GPR78 (Ranganathan et al., 2006), HSP47 (Chern et al., 2020), and HSP90 (Azad et al., 2015). However, ER stress can also be an important target for cancer therapy. Previous studies have shown that cytotoxic compounds targeting the ER are generally more selective for cancer cells than for noncancer cells (King and Wilson, 2020). In melanoma cells, direct knockdown of XBP1, an essential UPR gene, enhances the effect of immune checkpoint (ICP) inhibitors (Chen and Cubillos-Ruiz, 2021). The aforementioned results suggest that studies of ER stress-related genes may be of great value in predicting the prognosis of GAC patients and could serve as potential therapeutic targets.

In this research, we constructed and validated an ER stress-related signature that not only predicts the prognosis of GAC patients but also distinguishes the immune infiltration characteristics of GAC. Our study provides a novel perspective for future studies of ER stress and GAC.

## Materials and methods

### Data acquisition

GAC patient mRNA expression profiles and corresponding clinical information were downloaded from The Cancer Genome Atlas (TCGA). Tumor samples with complete clinical information, including M, N, and T stage, TNM stage, and survival duration longer than 1 month, were included in the study (a total of 290 samples). mRNA expression values were converted to TPM (transcripts per kilobase of exon model per million mapped reads) values. GSE84433 was profiled on an Illumina HumanHT-12 V3.0 Expression BeadChip, including 357 gastric cancer samples, which resulted in 355 samples in our investigation, as 2 samples were from patients whose survival time was less than 1 month (Table 1). TCGA dataset was utilized as a training cohort to construct the ER stress prognosis model, and the GSE84433 dataset was used as a validation cohort to verify the signature’s prediction potential. Somatic mutation data (MuTect2 Variant Aggregation and Masking) were downloaded from UCSC Xena. Copy number variation (CNV) data were downloaded from TCGA database using the “TCGAbiolinks” R package.

**TABLE 1:** Clinical information of tumor samples included in this study.

Clinical characteristic	TCGA cohort	GSE84433
Total cases	290 (100%)	355 (100%)
Event		
Dead	121 (41.7%)	172 (48.5%)
Alive	169 (58.3%)	183 (51.5%)
Sex		
Male	181 (62.4%)	240 (67.6%)
Female	109 (37.6%)	115 (33.4%)
Race		
Asian	59 (20.3%)	—
Black or African American	9 (3.1%)	—
Native Hawaiian or other Pacific islanders	1 (0.3%)	—
Not reported	40 (13.8%)	—
White	181 (62.4%)	—
Age		
65≤	133 (45.9%)	240 (67.6%)
65>	157 (54.1%)	115 (33.4%)
M		
M0	262 (90.3%)	—
M1	19 (6.6%)	—
MX	9 (3.1%)	—
N		
N0	89 (30.7%)	71 (20.0%)
N1	76 (26.2%)	154 (43.4%)
N2	62 (21.4%)	99 (27.9%)
N3	59 (20.3%)	31 (8.7%)
NX	4 (1.4%)	—
T		
T1	12 (4.1%)	11 (3.1%)
T2	64 (22.1%)	35 (9.9%)
T3	142 (49.0%)	67 (18.9%)
T4	72 (24.8%)	242 (68.2%)
TNM Stage		
Stage I	40 (13.8%)	—
Stage II	98 (33.8%)	—
Stage III	122 (42.1%)	—
Stage IV	30 (10.3%)	—

### Construction and validation of the signature

The phrase “endoplasmic reticulum stress” was used as a keyword to search for related genes in the Molecular Signatures Database, eventually obtaining 16 gene sets (Supplementary Material S1). After eliminating duplicates, 465 genes present in the training and validation cohorts were included in the next step of the analysis. Univariate Cox regression analysis was used to identify ER stress-related genes associated with OS. Genes with a *p*-value less than 0.05 were considered significant prognostic signatures. Least absolute shrinkage and selection operator (LASSO) Cox regression analysis was used to construct a prognostic signature based on the identified prognostic ER stress-related genes using the “glmnet” R package. Next, 10-fold cross-validation was employed to determine the penalization parameter (*λ*). Finally, those genes with nonzero coefficients were chosen to develop the signature. The patient risk score was computed using the following equation:

$$\begin{array}{l} Riskscore=\overset{n}{\underset{i=1}{\sum }}coef_{i}\times expression_{i}. \end{array}$$ (1)

The median risk score was used to divide patients into high-risk and low-risk groups in both datasets. To evaluate the predictive power of the signature, the “survival” R package was used to draw survival curves between the low-risk and high-risk groups. In addition, time-dependent receiver operating characteristic (ROC) curves were applied to evaluate the prognostic value of the risk score for OS using the “timeROC” R package The “survival” R package was also used to perform univariate and multivariate Cox regression analyses to verify the risk score’s independent prediction power for other clinicopathological variables. The relationship between clinicopathological characteristics and risk scores was analyzed using Kaplan–Meier curves.

### Construction of the predictive nomogram

The “rms” R package was used to generate a nomogram to predict the 1-, 3-, and 5-year survival rates of GAC patients in the training cohort by age, M stage, N stage, T stage, TNM stage, and risk score. The calibration curves were used to evaluate the predictive power of our constructed nomogram.

### Gene set enrichment analysis

Gene set enrichment analysis (GSEA) (Subramanian et al., 2005) was utilized to identify molecular processes and functional pathways that differed between the high-risk and low-risk groups in the training cohort. The hallmark gene set and KEGG gene set were retrieved from the Molecular Signatures Database. Statistical significance was defined as NOM *p*-value <0.05, FDR <0.25, and |NES| > 1.

### Immune infiltration analysis

To measure the correlations between our signature and tumor immune cell infiltration, CIBERSORT (Newman et al., 2015) was used to quantify 22 types of tumor-infiltrating immune cells in each TCGA sample. The Wilcoxon test was used to compare immune infiltration and function in the high-risk group to those in the low-risk group. ESTIMATE (Yoshihara et al., 2013) was used to estimate the tumor purity with the “estimate” R package.

### Somatic mutation and ICP analyses

TCGA mutation data were analyzed using “maftools” to explore the differences in GAC patient somatic mutations and tumor mutation burden (TMB) between the high-risk and low-risk groups. To assess the efficacy of the signature in immunotherapy, boxplots were constructed to analyze the expression of common ICPs, including PDCD1 (PD1), CD274 (PD-L1), LAG3, CTLA4, and PDCD1LG2 (PD-L2).

### CNV analysis

GISTIC2.0 (Mermel et al., 2011) was used to identify regions with significant amplifications or deletions. The threshold for amplification and deletion scores was ˃ 0.1, and the q-value threshold was <0.05.

### Statistical analyses

Computational and statistical analyses in this study were conducted using R (version 4.1.1). The log-rank test was used to analyze survival. For quantitative data, statistical significance for comparisons between two groups or more than two groups was estimated using the Wilcoxon test. Statistical significance was defined as a *p*-value <0.05.

## Results

### Construction and evaluation of the prognostic signature based on ER stress-related genes

**FIGURE 1:**
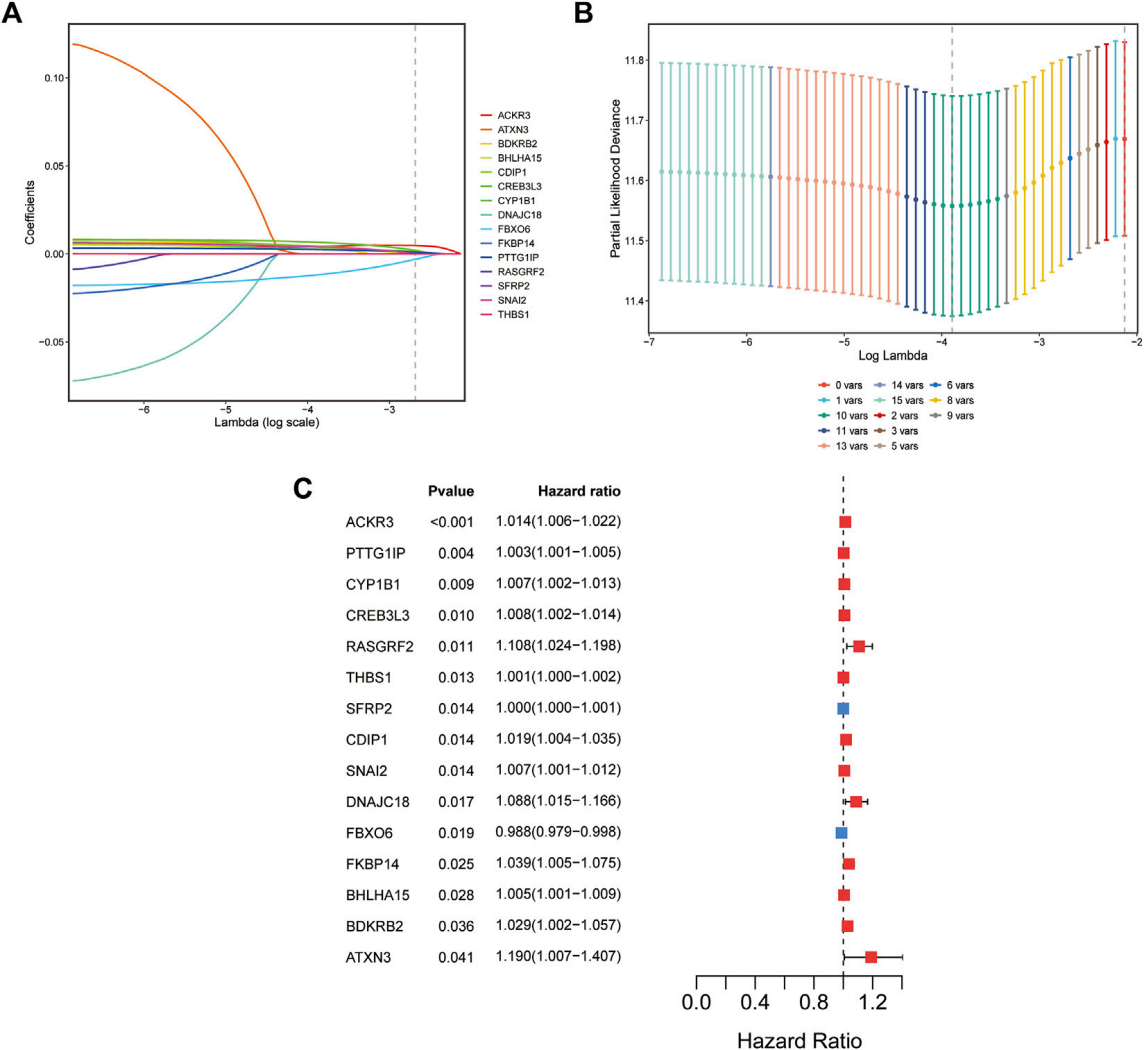
Establishment of the ER stress-related prognostic signature. **(A)** Coefficients of the LASSO regression model for 15 genes in the training cohort (TCGA cohort). **(B)** Cross-validation of 15-gene LASSO regression results. **(C)** Forest plot of univariate Cox regression analysis of 15 ER stress-associated genes.

**FIGURE 2:**
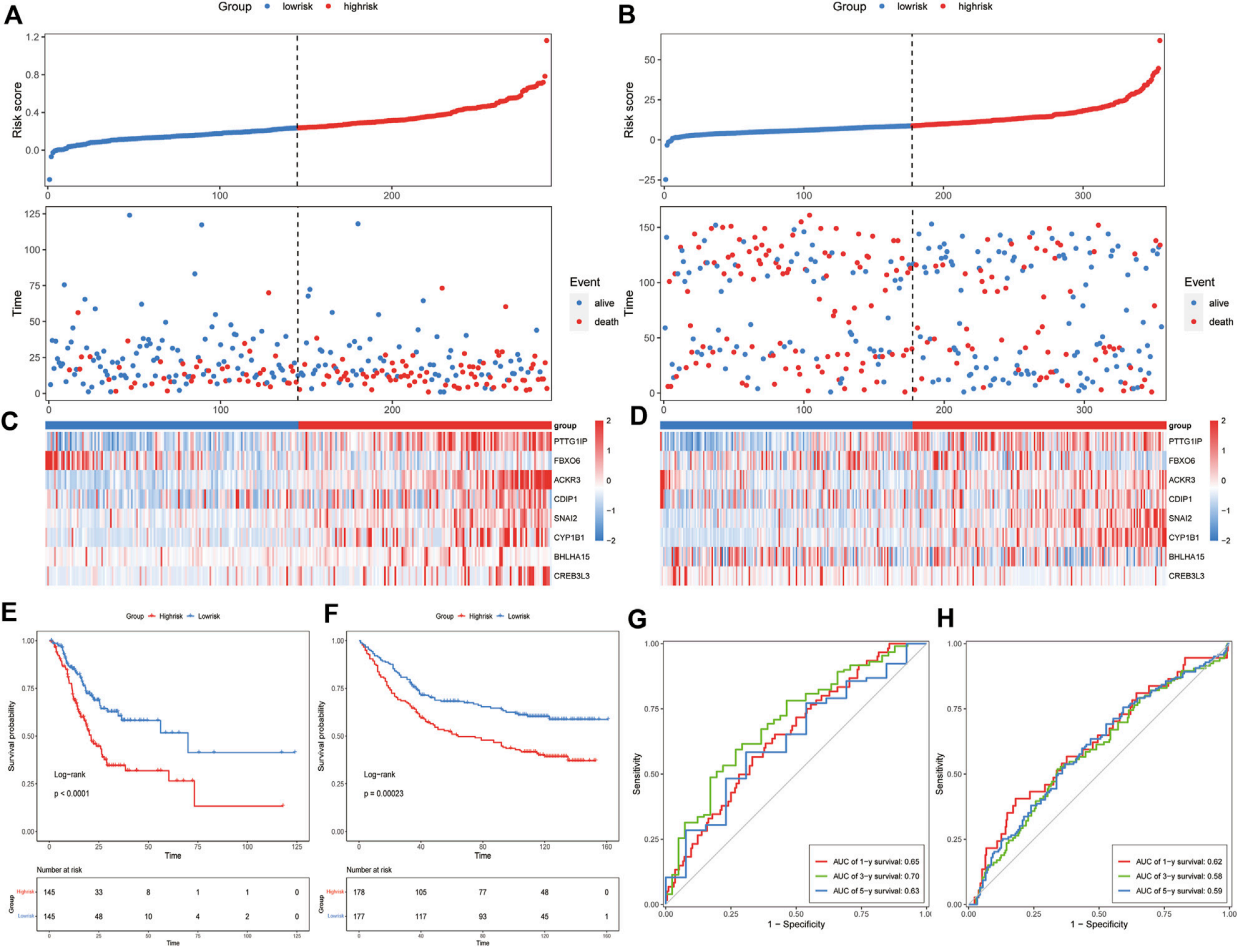
Prognostic value of the ER stress-related signature in the training and validation datasets. Risk score distribution and survival overview of each GAC patient **(A)** in the training cohort and **(B)** in the validation cohort. The heatmap of the eight genes’ expression **(C)** in the training cohort and **(D)** in the validation cohort. Survival analysis of the signature **(E)** in the training cohort and **(F)** in the validation cohort. ROC curves of the signature using the 8-gene signature **(G)** in the training cohort and **(H)** in the validation cohort.

Univariate Cox regression analysis was applied to identify prognostic ER stress-related genes in the training cohort. A total of 15 prognostic genes associated with ER stress (Figure 1C) were used for subsequent signature construction in the training cohort. A penalty parameter was selected based on the results of LASSO regression and 10-fold cross-validation (Figures 1A,B). A total of eight genes with nonzero coefficients associated with that penalty parameter were obtained to construct the risk score formula (Supplementary Material S2). The risk score for each patient in the training and validation sets was calculated according to the developed formula. The distributions of risk scores, outcome events, and survival time for each patient in both cohorts are shown in scatter plots in Figures 2A, C. These results also suggested a trend in which patients with GAC in the high-risk group died earlier than those in the low-risk group in both the training and validation sets. Additionally, the expression of the eight genes in both datasets is shown using a heatmap (Figures 2B, D). To evaluate the reliability of the signature, Kaplan–Meier survival analyses were carried out on both cohorts, which revealed that patients in the low-risk group experienced a higher overall survival rate (Figures 2E, F). Analysis of time ROC curves revealed area under the curve values for 1-, 3-, and 5-year OS of 0.65, 0.70, and 0.63 and 0.62, 0.58, and 0.59 in the training cohort and validation cohort, respectively, which demonstrated a good predictive accuracy of the signature (Figures 2G, H).

### Independent prognostic value of the ER stress-related signature

**FIGURE 3:**
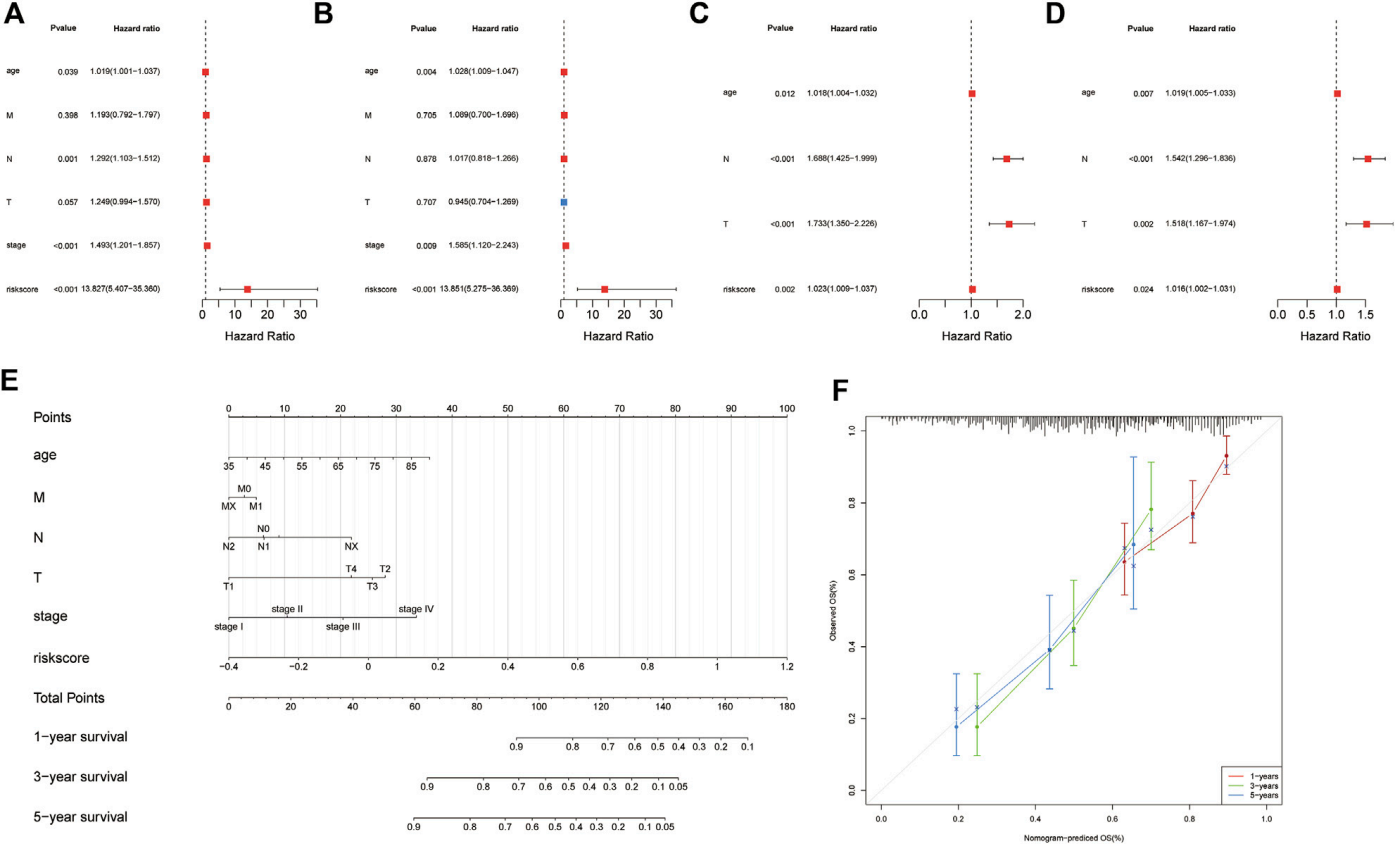
Identification of independent prognostic factors and establishing the nomogram. Forest plot for univariate Cox regression analysis of clinical information and risk scores **(A)** in the training cohort and **(B)** validation cohort. Forest plot for multivariate Cox regression analysis of clinical information and risk scores **(C)** in the training cohort and **(D)** validation cohort. **(E)** Patients’ risk scores and other clinical information from TCGA dataset were used to construct nomograms to predict the prognosis of patients with GAC at 1 year, 3 years, and 5 years. **(F)** Calibration plot of the nomogram in TCGA cohort.

To determine whether the signature could be an independent prognostic factor, univariate and multivariate Cox regression analyses were conducted among the clinical features and risk scores in both cohorts. The risk score was an independent prognostic factor in both the training and validation cohorts, according to the results of univariate Cox regression (training cohort: HR = 13.827, 95% CI = 5.407-35.36, and *p* = 4.19E-08; Validation cohort: HR = 1.0227, 95% CI = 1.009-1.037, and *p* = 0.00155448) (Figures 3A,C). In the multivariate Cox regression analysis, the risk score remained an independent predictor of OS (training cohort: HR = 13.8514, 95% CI = 5.2754-36.3691, and *p* = 9.47E-08; validation cohort: HR = 1.0164, 95% CI = 1.0021–1.0308, and *p* = 0.024195032) (Figures 3B,D). The results of the Cox regression analyses are shown in Supplementary Material S3.

### Construction and validation of the nomogram

To predict the survival probabilities of patients with GAC, a nomogram was constructed integrating risk score, age, M stage, N stage, T stage, and TNM stage (Figure 3E). The calibration chart shows the accuracy of the assessment for the prediction nomogram (Figure 3F). The nomogram performed well when predicting the probability of patient survival at 1, 3, and 5 years.

### The ER stress-related signature is associated with clinicopathological features

**FIGURE 4:**
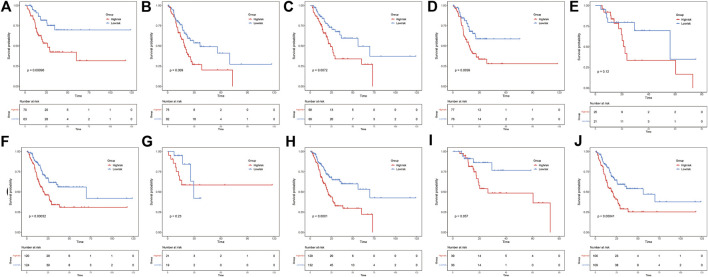
ER stress risk score can distinguish different clinicopathological features of gastric adenocarcinoma. Patients aged **(A)** ≤65 years and **(B)** > 65 years. Patients with **(C)** MX–M0 and **(D)** M1. Patients with **(E)** NX–N1 and **(F)** N2–N3. Patients with **(G)** T1–T2 and **(H)** T3–T4. Patients with **(I)** Stage I–Stage II and **(J)** Stage III–Stage IV.

To investigate the relationship between various clinicopathological characteristics and prognostic models, patients were divided into the following groups: age ≤65 years and age >65 years (Figures 4A,B), MX–M0 and M1 (Figures 4C,D), NX-N1 and N2-N3 (Figures 4E,F), T1–T2 and T3–T4 (Figures 4G,H), and Stage I–Stage II and Stage III–Stage IV (Figures 4I,J). The Kaplan–Meier curve analyses showed that the prognosis of the low-risk group was better than that of the control group, except for patients in the MX–M0, T1–T2, and Stage I–Stage II groups. This may be related to the fact that most patients were diagnosed with GAC at a late stage, resulting in a relatively small number of early-stage patients in the dataset (Digklia and Wagner, 2016).

### GSEA between different risk groups

**FIGURE 5:**
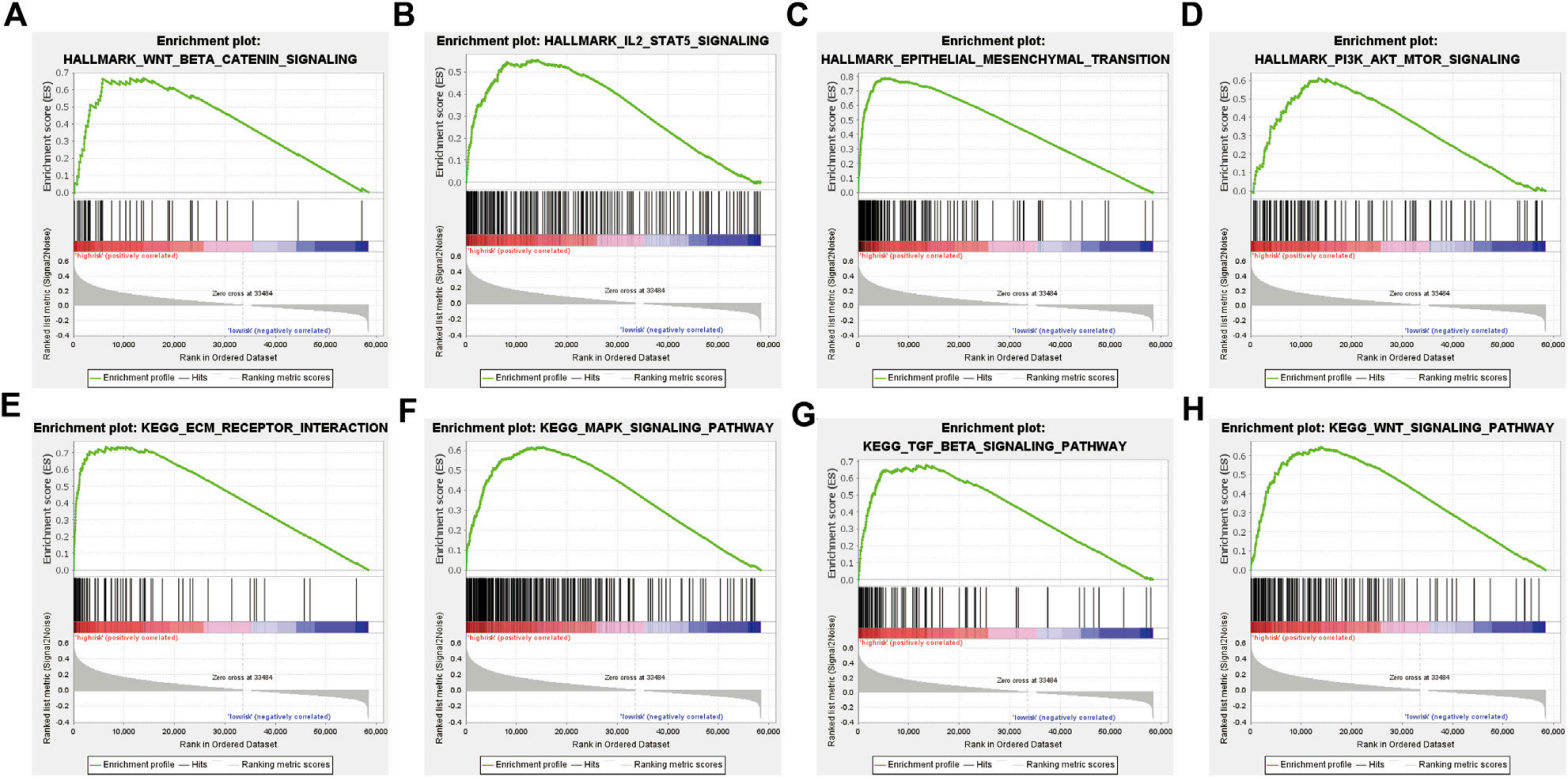
GSEA of the TCGA cohort. **(A–D)** Hallmark gene set enriched in the high-risk group. **(E–H)** KEGG pathways enriched in the high-risk group.

To investigate the potential function and significant pathway changes associated with the signature, the expression matrices of the different risk groups were imported and examined by GSEA. The analysis of the enrichment of the hallmark gene set indicated that many pathways closely related to tumor progression were activated in the high-risk group, such as epithelial–mesenchymal transition, IL2-STAT5 signaling, PI3K-AKT-MTOR signaling, and WNT β-catenin signaling (Figures 5A–D). In addition, the results of KEGG enrichment analysis showed that many pathways were enriched in the high-risk group, including ECM–receptor interaction, MAPK signaling pathway, TGF beta signaling pathway, and WNT signaling pathway (Figures 5E–H). All of the enrichment analysis results are shown in Supplementary Material S4. Previous studies have shown that oncogenic signaling pathways frequently activated in tumor cells, including the MAPK and WNT/β-catenin pathways, can induce tumor cells to produce immunosuppressive factors, leading to immunosuppression in the tumor microenvironment (Yaguchi et al., 2011). Similarly, the activity of most immune cells is influenced to some extent by the PI3K-AKT-MTOR signaling pathway (O'Donnell et al., 2018). These results suggest a positive correlation between the ER stress risk score and the malignancy of tumors in patients with GAC.

### The ER stress-related signature is correlated with the tumor immune status

**FIGURE 6:**
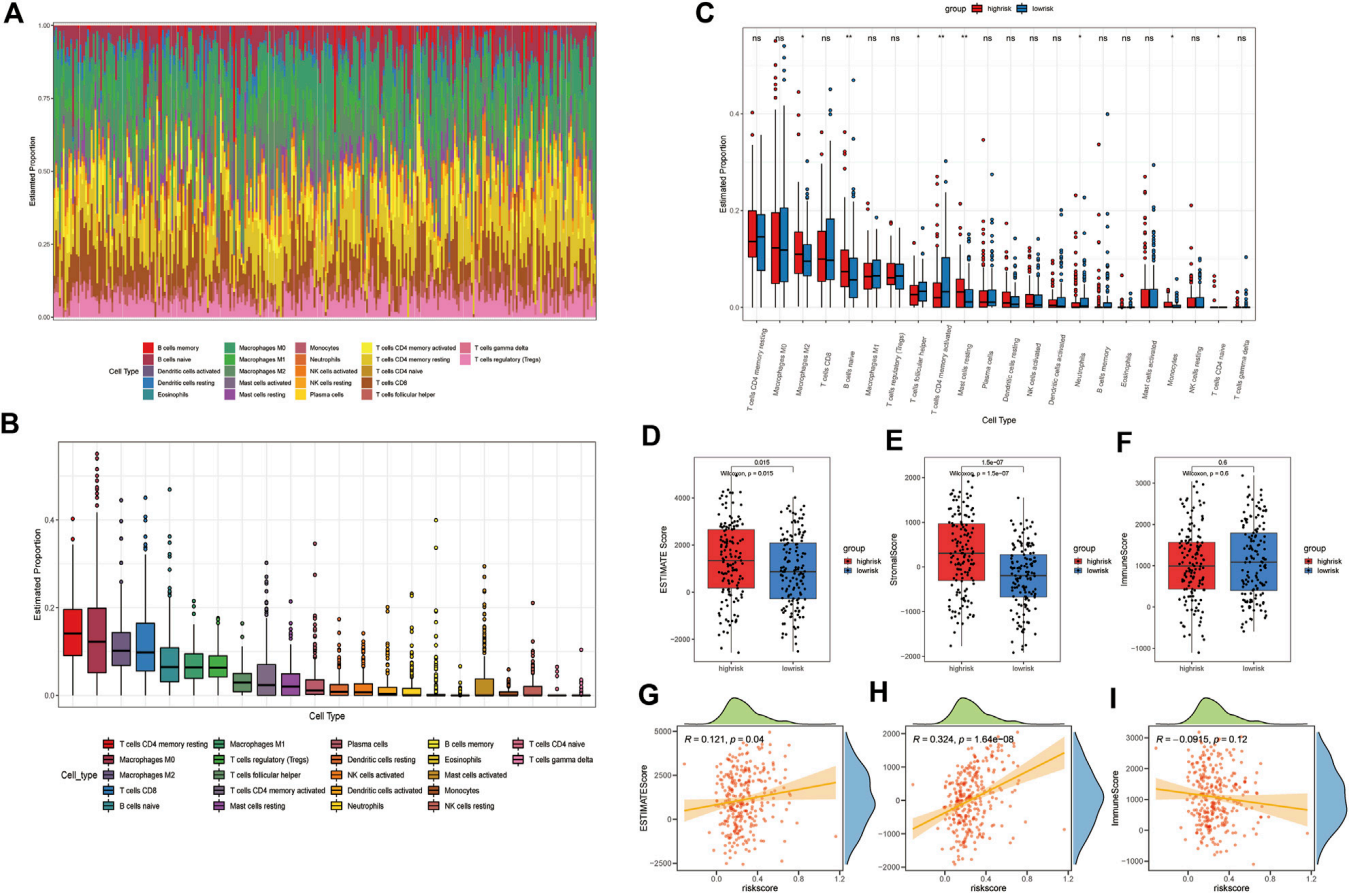
Immune landscape between different groups of GAC patients. **(A)** Identity plot of 22 types of immune cells per patient. **(B)** Proportion of 22 types of immune cells. **(C)** Boxplot of the proportion of 22 immune-infiltrating cells in the high- and low-risk groups (*: *p*-value <0.05, **: *p*-value <0.01, and ***: *p*-value <0.001). Boxplots of **(D)** estimated score, **(E)** stromal score, and **(F)** immune score, calculated by ESTIMATE algorithm in high- and low-risk groups. The correlation plot of the risk score with the **(G)** estimated score, **(H)** stromal score, and **(I)** immunization score.

According to the GSEA results, we hypothesized that our risk signature can detect differences in the immune infiltration status of patients. The CIBERSORT algorithm was then used to investigate the relationship between the ER stress model and the tumor immune microenvironment. First, an identity plot and a boxplot were used to roughly determine the distribution of 22 immune cells in GAC patients, as calculated using CIBERSORT (Figures 6A, B). Second, to assess the differences in the proportions of the 22 types of immune cells between different risk groups, we constructed a boxplot, which demonstrated that the proportions of M2 macrophages, naïve B cells, resting mast cells, and monocytes were higher in the high-risk group than in the low-risk group. The proportions of follicular helper T cells, activated CD4 memory T cells, and neutrophils were lower in the high-risk group than in the low-risk group (Figure 6C). The ESTIMATE algorithm was then used to compute the estimated score, stromal score, and immune score in both groups. The results showed that the estimated score and stromal score were significantly higher in the high-risk group than in the low-risk group. The immune score was lower in the high-risk group than in the low-risk group, but the difference was not statistically significant (Figures 6D–F). In addition, patients’ risk scores exhibited a significant positive correlation with the ESTIMATE and stromal scores, but there was no significant correlation with immune scores. These results not only suggested that immune cell infiltration may have a significant impact on the prognosis of patients with GAC but also suggested the efficacy of the signature.

### Tumor mutation and immune checkpoint landscape in GAC patients

**FIGURE 7:**
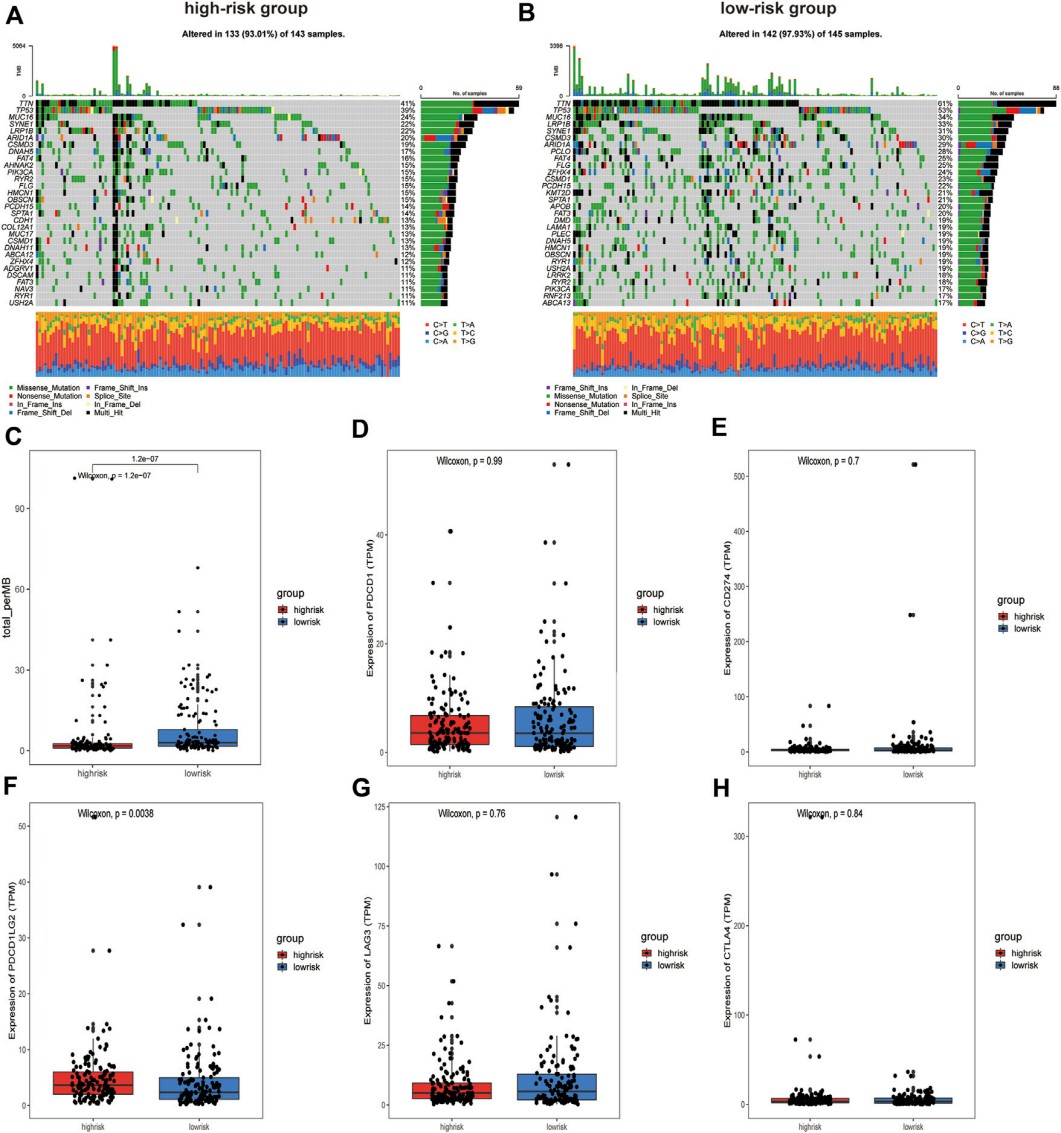
Analysis of somatic mutations and Immune checkpoint expression in patients in high and low-risk groups. **(A)** Oncoplot of the top 30 mutation genes in the high-risk group and **(B)** in the low-risk group. **(C)** Box plot of TMB values for patients in the different groups. **(D)** PDCD1, **(E)** CD274, **(F)** PDCD1LG2, **(G)** LAG3, and **(H)** CTLA4 expression between the high-risk and low‐risk groups.

To evaluate differences in tumor mutation profiles between different risk groups, the top 30 mutated genes in the high- and low-risk groups in terms of mutation frequency were displayed using an oncoplot (Figures 7A,B). Interestingly, the top five genes in terms of mutation frequency were the same for patients in the high-risk and low-risk groups. TMB has been found in certain studies to be a useful tool for identifying individuals with a variety of cancers who are better candidates for immunotherapy (Chan et al., 2019). To determine whether our signature can identify patients who are more suitable for immunotherapy, the TMB of patients was calculated using maftools. The results showed that the TMB in the low-risk group was significantly higher than that in the high-risk group, suggesting that patients in the low-risk group may have a better response to immunotherapy (Figure 7C). We then compared the expression levels of ICPs in the high-risk and low-risk groups, and the results showed that ICP molecules such as PDCD1 (Figure 7D), CD274 (Figure 7E), LAG3 (Figure 7G), and CTLA4 (Figure 7H) were expressed at similar levels in both groups. Only the expression of PDCD1LG2 (PDL2) differed significantly between the high-risk and low-risk groups (Figure 7F).

### CNV profile in GAC patients

**FIGURE 8:**
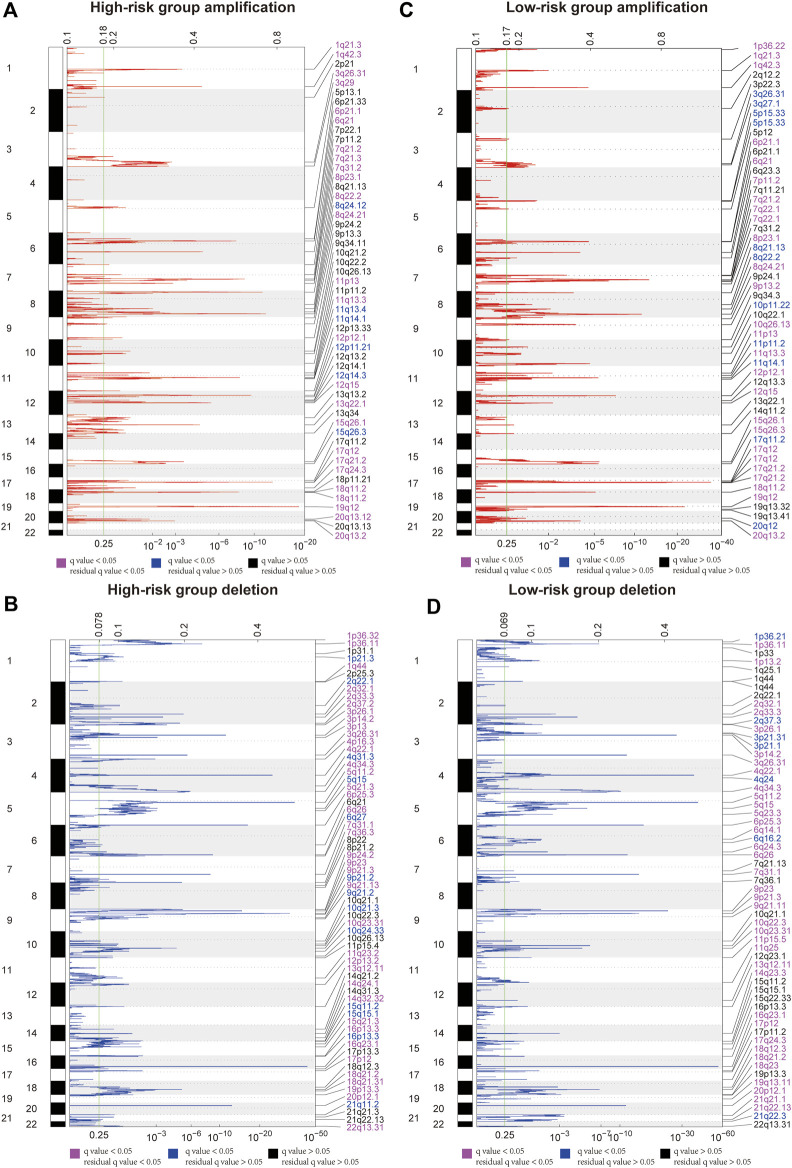
Landscape of copy number variants in high- and low-risk groups. **(A)** Amplification in the high-risk group. **(B)** Deletion in the high-risk group. **(C)** Amplification in the low-risk group. **(D)** Deletion in the low-risk group (dark pink: residual q-value and q-value below 0.05, blue: only q-value below 0.05, and black: residual q-value and q-value greater than 0.05).

**FIGURE 9:**
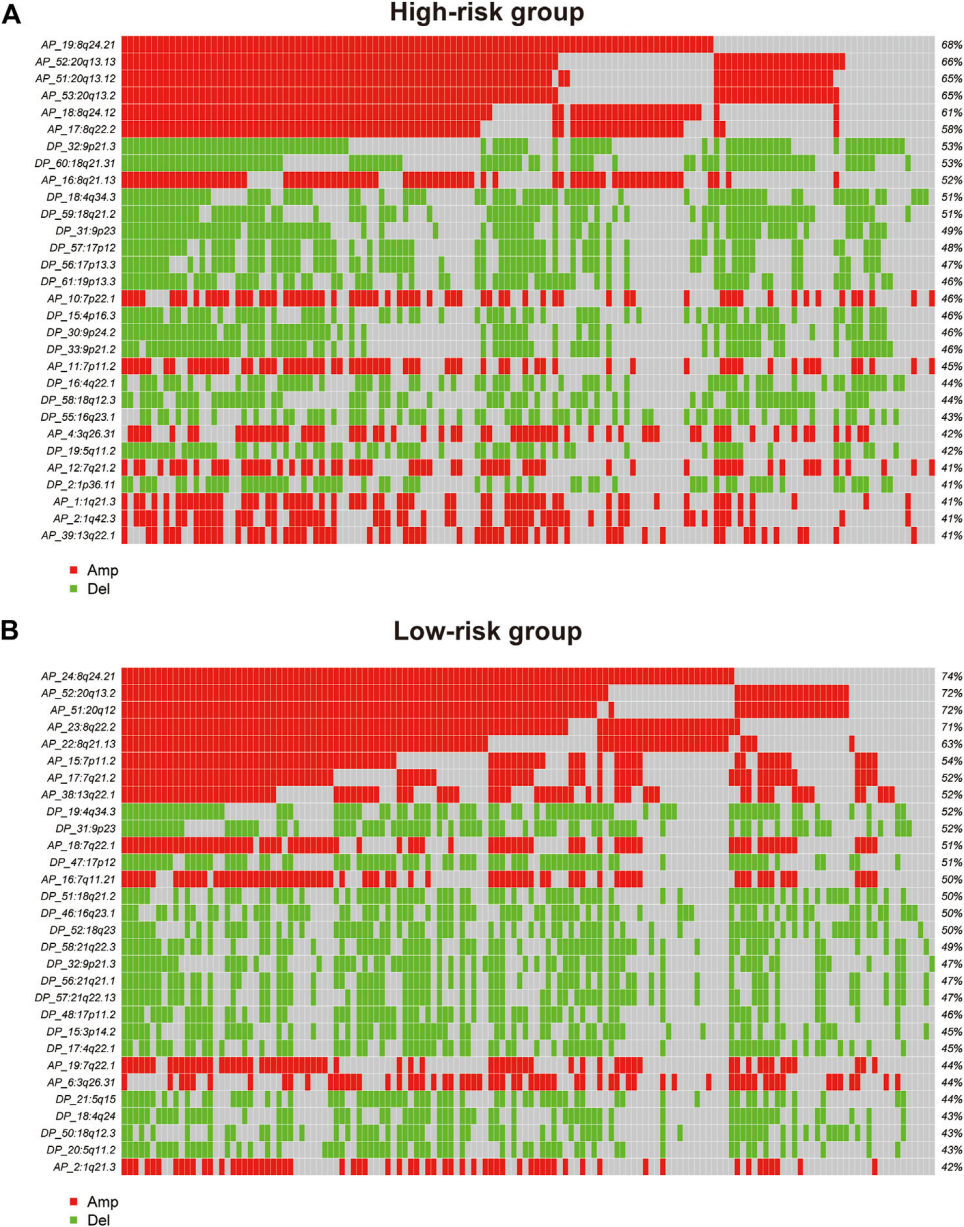
Oncoplot shows the top 30 regions of amplification and deletion frequencies for **(A)** the high-risk and **(B)** low-risk groups.

GISTIC2.0 was used to detect CNVs between the different risk groups. For all significant amplification and deletion regions, a peak region with the greatest amplitude and frequency of change was detected, and the corresponding gene and chromosome regions are shown in Supplementary Material S5. A total of 53 amplified regions and 66 deletion regions were found in the high-risk group (Figures 8A,B). A total of 52 amplified regions and 59 deletion regions were detected in the low-risk group (Figures 8C,D). In the CNV analysis, the five most significantly amplified and deleted regions in the high-risk group were 19q12, 8p23.1, 8q24.21, 17q12, and 12p12.1 and 16q23.1, 5q11.2, 9p21.3, 4q22.1, and 6p25.3, respectively (Table 2). Similarly, in the low-risk group, significant amplification and deletion regions were 17q12, 19q12, 8q24.21, 7q21.2, and 12p12.1 and 16q23.1, 5q11.2, 4q22.1, 3p14.2, and 9p23, respectively (Table 3). To investigate the frequency of amplifications and deletions in the genome of GAC patients, we imported the GISTIC results into maftools. An oncoplot plot shows the top 30 regions exhibiting differences in copy number frequency between GAC patient groups (Figures 9A,B). The regions with the highest frequencies of copy number amplifications and deletions in the high-risk group were 8q24.21 and 9p21.3, respectively. The regions with the highest frequencies of copy number amplifications and deletions in the low-risk group were 8q24.21 and 4q34.3, respectively.

**TABLE 2:** Most significant amplification/deletion region in the high-risk group.

Cytoband	CNV type	q-value	Residual q-value	Wide peak boundary	Genes in the wide peak
19q12	Amp	3.27E-19	3.27E-19	chr19:29774394-29907229	CCNE1
8p23.1	Amp	5.98E-12	5.98E-12	chr8:11739598-11786293	GATA4, NEIL2, and C8orf49
8q24.21	Amp	1.73E-12	1.26E-11	chr8:127701853-127712180	CASC11
17q12	Amp	1.21E-13	2.64E-11	chr17:39698255-39720948	ERBB2
12p12.1	Amp	2.51E-10	3.46E-10	chr12:25186964-25361662	KRAS, CASC1, and ETFRF1
16q23.1	Del	1.58E-44	1.58E-44	chr16:78095161-79593873	WWOX
5q11.2	Del	2.65E-36	4.18E-36	chr5:58964471-60492158	PDE4D, PART1, and MIR582
9p21.3	Del	1.31E-33	2.60E-31	chr9:21865499-21997723	CDKN2A and CDKN2A-AS1
4q22.1	Del	2.33E-25	2.33E-25	chr4:90227129-92262630	CCSER1
6p25.3	Del	5.05E-17	5.05E-17	chr6:1608602-2252191	FOXC1 and GMDS

CNV, copy number variation; Amp, copy number amplification; Del, copy number deletion.

**TABLE 3:** Most significant amplification/deletion region in the low-risk group.

Cytoband	CNV type	q-value	Residual q value	Wide peak boundary	Genes in the wide peak
17q12	Amp	1.45E-34	3.90E-25	chr17:39692293-39747957	ERBB2, GRB7, MIEN1, and MIR4728
19q12	Amp	3.23E-23	3.23E-23	chr19:29815851-29825968	CCNE1
8q24.21	Amp	5.68E-12	1.57E-11	chr8:127214429-127226866	CCAT1
7q21.2	Amp	1.83E-13	1.98E-11	chr7:92525796-92928684	CDK6, PEX1, RBM48, FAM133B, and LOC101927497
12p12.1	Amp	5.17E-08	5.17E-08	chr12:25177518-25437858	KRAS, CASC1, and ETFRF1
16q23.1	Del	1.58E-56	1.58E-56	chr16:78095161-79593873	WWOX
5q11.2	Del	3.71E-40	8.78E-40	chr5:58964471-60492158	PDE4D, PART1, and MIR582
4q22.1	Del	1.29E-37	2.17E-37	chr4:90227129-92262630	CCSER1
3p14.2	Del	5.19E-28	4.22E-27	chr3:59717096-61040678	FHIT and MIR548BB
9p23	Del	3.67E-24	3.67E-24	chr9:8310705-10619051	PTPRD, PTPRD-AS1, and LOC105375972

CNV, copy number variation; Amp, copy number amplification; Del, copy number deletion.

## Discussion

ER stress is reportedly involved in tumor progression and exhibits great potential as a target in cancer treatment (Marciniak et al., 2022). On the one hand, the ER stress-induced UPR can make tumor cells more tolerant to an unfavorable environment. Tumor cells can transmit their ER stress state to certain immune cells to suppress antitumor immunity (Mahadevan et al., 2011; Lee et al., 2014). On the other hand, excessive ER stress can lead to cell death (Oakes and Papa, 2015). The effect of ER stress on tumor cells is dependent on the expression of ER stress-related genes. Moreover, previous studies reported that the overexpression of some ER stress-related genes is correlated with poor prognosis in patients with several types of cancer, including breast cancer (Chen et al., 2014), endometrial cancer (Matsuo et al., 2013), and malignant melanoma (Shimizu et al., 2017). However, the prognostic value of ER stress-related genes in GAC remains to be fully elucidated.

In this study, a signature of eight ER stress-related genes was established based on TCGA cohort and validated in the GEO cohort. These eight genes include PTTG1IP, FBXO6, ACKR3, CDIP1, SNAI2, CYP1B1, BHLHA15, and CREB3L3. According to univariate Cox analysis, higher expression of PTTG1IP, ACKR3, CDIP1, SNAI2, CYP1B1, BHLHA15, and CREB3L3 is an unfavorable factor for OS in GAC patients, whereas higher expression of FBXO6 is a favorable factor.

Previous studies have reported the roles of the aforementioned genes. For instance, PTTG1IP (PTTG1-binding factor, PBF/PTTG1IP) is a proto-oncogene. In thyroid cells, PTTG1IP specifically binds to p53 and significantly inhibits the transactivation of responsive promoters, and PTTG1IP reduces the stability of p53 (Read et al., 2014). According to another report, p53 participates in the regulation of mitochondrial and ER interactions, and the knockdown of p53 reduces ER stress-induced injury in mouse cardiomyocytes by protecting mitochondria (Chen et al., 2019). Interestingly, a mutant of PTTG1IP, C51R, was reported to be mainly confined to the endoplasmic reticulum (Imruetaicharoenchoke et al., 2017). PTTG1IP may not be directly related to ER stress, but they may be indirectly involved in ER stress processes due to their close interaction with p53.

SNAI2 (Snail family transcriptional repressor 2) is a member of the Snail family of proteins and plays a crucial role in the developmental process (Cobaleda et al., 2007). In ER stress-activated HEK293T cells, IRE1-XBP1 signaling pathway activation can upregulate the expression of some EMT-TFs including SNAI2 (Cuevas et al., 2017). Moreover, SNAI2 induces MDM2 expression to promote p53 degradation in colon cancer cells. It has also been shown that the loss of p53 in tumors activates the IRE1α/XBP1 pathway to enhance protein folding and secretion (Namba et al., 2015).

FBXO6 (F-box protein 6, FBG2) is a substrate recognition component of certain SCF (SKP1, CUL1, and F-box protein)-type E3 ubiquitin ligases involved in the degradation of ER-associated proteins, and its role in cancer is highly complex. Previous studies have shown that FBXO6 inhibits cadmium-induced ER stress and reduces cell death induced by subsequent c-Jun N-terminal kinase 1 (JNK1) activation (Du et al., 2014). Furthermore, it has been demonstrated that FBXO6 inhibits Chk1 activation in non-small cell lung cancer, increasing cisplatin sensitivity (Cai et al., 2019).

ACKR3 (atypical chemokine receptor 3, CXCR7) is an atypical chemokine receptor that binds to chemokines to control chemokine levels and localization. ACKR3 is reportedly involved in protecting cardiomyocytes from palmitate-induced ER stress and apoptosis induced by SDF-1β (Zhao et al., 2013).

CDIP1 is a pro-apoptotic gene. Previous studies have shown that the loss of CDIP1 blocks ER stress-induced apoptosis (Namba et al., 2013). CYP1B1 encodes an enzyme that is a member of the cytochrome (CYP) P450 family. Human granular lutein cells in a state of ER stress can increase the expression of AHR and ARNT, increasing CYP1B1 expression and activity (Kunitomi et al., 2021). Atrazine activates nuclear xenobiotic receptor responses that disrupt CYP P450 homeostasis and CYP isoform transcription, including CYP1B1, triggering inflammatory injury in the heart induced by pathways such as ER stress (Li X.-N. et al., 2018).

BHLHA15 is also known as MSIT1. BHLHA15 expression is downregulated in pancreatic cancer tissues, whereas overexpression of BHLHA15 inhibits the proliferation, migration, and invasion of human pancreatic cancer cells (Li X. et al., 2018). In a state of ER stress, BHLHA15 induced by XBP1 can enhance the UPR by synergizing the expression of genes encoding multiple secreted proteins (Hess et al., 2016).

CREB3L3 (CAMP-responsive element binding protein 3 like 3) is a transmembrane transport factor (Llarena et al., 2010). In hepatocytes, proinflammatory cytokines can induce cleavage of CREB3L3 and activate the acute phase response and UPR (Zhang et al., 2006).

Based on the 8-gene signature described here, patients who had low-risk scores had a better prognosis and longer survival time in the training and validation cohorts. The results of Cox regression analyses suggested that our risk score is an independent prognostic factor. Time-ROC curve analyses revealed that the risk signature predicted better short- and long-term survival for GAC patients in both datasets. In brief, the aforementioned results demonstrated the good predictive effect of our ER stress-related signature.

According to the GSEA results, immune-related pathways were upregulated in the high-risk group, indicating that our ER stress signature is potentially related to the tumor immune microenvironment in GAC. Thus, we performed ESTIMATE and CIBERSORT analyses to determine the relationship between our signature and immune infiltration. The high-risk group had higher estimate scores and stromal scores, suggesting that these scores are associated with poor prognosis in GAC. In addition, a significant increase in the proportion of M2 macrophages in patients in the high-risk group was observed. Previous studies reported that M2 macrophages recruited by tumor-initiating cells can promote the immune escape of tumor-initiating cells (Guo et al., 2017). Similarly, polarized M2 macrophages induced by stromal cells reportedly promote metastasis of gastric cancer cells (Yamaguchi et al., 2016; Li et al., 2019). Interestingly, the aforementioned data suggest that higher proportions of M2 macrophages and higher stromal scores are associated with poor prognosis in patients with GAC. Meanwhile, the proportions of follicular helper T cells, activated CD4 memory T cells, and neutrophils in the high-risk group were significantly lower. Previous studies have shown that follicular helper T cells can restore antitumor responses in the immune microenvironment in a CD8+-dependent manner (Niogret et al., 2021). Neutrophils release a variety of cytokines that promote T-cell proliferation and cytokine synthesis to enhance the adaptive immune response (Tillack et al., 2012). Collectively, high-risk patients had a reduced proportion of immune cells and demonstrated a state of immunosuppression. These results confirm the promising ability of the ER stress-related model to differentiate the immune infiltration status of GAC patients and may provide clinical recommendations for treatment.

The accumulation of somatic mutations and other genetic alterations can damage cell division checkpoints, leading to abnormal cell proliferation and ultimately tumor formation (Iranzo et al., 2018). The TMB is broadly defined as the number of somatic mutations per megabase of the genomic sequence, and it is correlated with the response rate of different tumors to ICP inhibitors (Barroso-Sousa et al., 2020). To identify the potential mechanism of our signature, we analyzed the somatic mutation profile and expression of ICPs in GAC patients. Unexpectedly, low-risk GAC patients had a greater TMB, suggesting that low-risk patients may be more likely to benefit from immunotherapy. This result is consistent with the findings of Shao et al. (2021). Among the common ICPs, however, only the expression of PDL2 was significantly higher in the high-risk group. The affinity of PDL2 for PD1 was three times higher than that of PDL1 (Ohaegbulam et al., 2015). An anti-PD1 antibody can block the binding of both PDL1 and PDL2 to PD1, increasing the tumor cell-killing ability of T cells compared with an anti-PDL1 antibody (Zou et al., 2016). We thus hypothesized that the high-risk group may be better suited for treatments targeting PD1.

Somatic CNVs can be used to identify genomic regions involved in disease phenotypes (Shlien and Malkin, 2009). Interestingly, the top five copy number amplification regions in both risk groups overlapped at two regions: 8q24.21 and 20q13.2. Similarly, the copy number deletion regions had one overlapping region, namely, 9p23. Although 8q24.21 and 20q13.2 amplifications were detected in both risk groups, the peaks with the greatest variation and frequency detected in those regions and the genes corresponding to the peaks were different. The region in which CASC11 is located was the most significantly amplified region on 8q24.21 in the high-risk group. However, the gene detected in the same region in the low-risk group was CCAT1. The most amplified gene detected in the 20q13.2 region was LOC105372672 in the high-risk group, but in the low-risk group, ZNF217, TSHZ2, LOC101927770, and LOC105372672 were detected. These results suggested that this ER stress-related prognostic model is also able to distinguish copy number changes in patients with GAC.

Our study has some limitations. First, the relatively small dataset sourced from public databases limited the predictive effect of our constructed model. Second, the expression and prognostic predictive effects of these eight genes at the protein level need further evaluation. Third, the ability of our model as an early biomarker remains to be developed. In the future, we will work to improve the reliability and ability of our model as an early biomarker. Despite these limitations, our study still provides a landscape of ER stress in GAC that may be useful for future studies.

## Conclusion

We constructed an ER stress-related prognostic signature to predict the prognosis of GAC patients. The signature was closely related to the immune infiltration status of patients. These eight ER stress-related gene signatures are good predictors of prognosis for GAC patients and may provide new perspectives for clinical treatment.

## Data Availability

The original contributions presented in the study are included in the article/Supplementary Material; further inquiries can be directed to the corresponding authors.
