# Supplementary material for: Establishment of an endoplasmic reticulum stress-related signature predicting outcomes of gastric adenocarcinoma patients
Source: Front Genet. 2022 Sep 6;13:944105. doi: 10.3389/fgene.2022.944105 (PMC9486073; doi:10.3389/fgene.2022.944105)
Supplement: Supplementary file 1 [file DataSheet1.ZIP › Supplementary Material 2.docx]

Supplementary Table 2 **The coef of the 8 genes**

| **Gene** | **Coef** |
| --- | --- |
| PTTG1IP | 1.03E-03 |
| FBXO6 | -3.03E-03 |
| ACKR3 | 4.82E-03 |
| CDIP1 | 2.68E-05 |
| SNAI2 | 2.50E-10 |
| CYP1B1 | 1.34E-03 |
| BHLHA15 | 1.13E-10 |
| CREB3L3 | 2.07E-03 |
