# Supplementary material for: Establishment of an endoplasmic reticulum stress-related signature predicting outcomes of gastric adenocarcinoma patients
Source: Front Genet. 2022 Sep 6;13:944105. doi: 10.3389/fgene.2022.944105 (PMC9486073; doi:10.3389/fgene.2022.944105)
Supplement: Supplementary file 1 [file DataSheet1.ZIP › Supplementary Material 3.docx]

Supplementary Table 3.1 The result of univariate COX regression in TCGA GAC cohort.

|  | HR | 95L | 95H | p.value |
| --- | --- | --- | --- | --- |
| age | 1.0189 | 1.001 | 1.037 | 0.038695 |
| M | 1.1932 | 0.7922 | 1.797 | 0.39798 |
| N | 1.2918 | 1.103 | 1.512 | 0.001457 |
| T | 1.249 | 0.9935 | 1.57 | 0.05694 |
| stage | 1.4932 | 1.201 | 1.857 | 0.000309 |
| riskscore | 13.827 | 5.407 | 35.36 | 4.19E-08 |

Supplementary Table 3.2 The result of multivariate COX regression in TCGA GAC cohort.

|  | HR | 95L | 95H | Pvalue |
| --- | --- | --- | --- | --- |
| age | 1.0276 | 1.009 | 1.0467 | 0.003538 |
| M | 1.0891 | 0.6996 | 1.6957 | 0.705393 |
| N | 1.0173 | 0.8175 | 1.266 | 0.877679 |
| T | 0.9452 | 0.7041 | 1.2688 | 0.707482 |
| stage | 1.5848 | 1.1199 | 2.2428 | 0.009351 |
| riskscore | 13.8514 | 5.2754 | 36.3691 | 9.47E-08 |

Supplementary Table 3.3 The result of univariate COX regression in GSE84433 cohort.

|  | HR | 95L | 95H | p.value |
| --- | --- | --- | --- | --- |
| age | 1.0179 | 1.004 | 1.032 | 0.012303 |
| N | 1.6877 | 1.425 | 1.999 | 1.32E-09 |
| T | 1.7334 | 1.35 | 2.226 | 1.60E-05 |
| riskscore | 1.0227 | 1.009 | 1.037 | 0.001554 |

Supplementary Table 3.4 The result of univariate multivariate COX regression in GSE84433 cohort.

|  | HR | 95L | 95H | Pvalue |
| --- | --- | --- | --- | --- |
| age | 1.0193 | 1.0053 | 1.0334 | 0.006537 |
| N | 1.5425 | 1.2957 | 1.8364 | 1.11E-06 |
| T | 1.5178 | 1.1673 | 1.9736 | 0.001841 |
| riskscore | 1.0164 | 1.0021 | 1.0308 | 0.024195 |
